# Recurrent Catastrophic Ceramic Femoral Head Failure in Total Hip Arthroplasty

**DOI:** 10.1155/2014/837954

**Published:** 2014-06-04

**Authors:** S. M. M. Tai, L. Parker, N. J. de Roeck, J. A. Skinner

**Affiliations:** ^1^East and North Hertfordshire NHS Trust, Stevenage, SG1 4AB, UK; ^2^Royal National Orthopaedic Hospital, Stanmore, Middlesex, HA7 4LP, UK

## Abstract

Fracture of a modern ceramic head component in total hip replacement is an uncommon but catastrophic complication. Hence, the occurrence of a second ceramic head fracture in the same hip replacement of an individual represents a perishingly rare event. We present the case as a means of highlighting potential risk factors for ceramic head fracture and suggest possible management strategies in such cases.

## 1. Introduction

Early generations of ceramic bearing were frequently associated with ceramic fracture [1]. However, since their first use as a bearing surface in total hip arthroplasty, the biomechanical properties of ceramics have been vastly improved through hot isocratic pressing, laser marking, and proof-testing [2]. Hence, use of ceramic bearings in total hip arthroplasty, particularly in young and active patients, is seen as a reliable and safe choice, with low wear and minimal osteolysis demonstrable at follow-up [3]. We present a case report of a recurrent ceramic femoral component fracture in a patient, resulting in a second revision procedure. The majority of fractures involving modern ceramic bearings in total hip replacement involve fracture of the ceramic liner [4]; therefore, consecutive fractures of a ceramic head is an extremely rare event. This case may therefore be utilized to illustrate potential risk factors for ceramic component fracture and management strategies in such cases. The patient provided written consent for the use of her case.

## 2. A Case Report

A 25-year-old woman underwent a left THR for the sequelae of developmental dysplasia of the hip. The patient had previously undergone surgery on the ipsilateral side, comprising a femoral osteotomy and subsequent shelf procedure. The primary hip replacement was performed through a posterior approach using an uncemented Corail titanium stem with a Biolox Forte alumina ceramic 28 mm, +1.5 neck length, 12/14 tapered cone head (DePuy/Johnson and Johnson, Leeds, United Kingdom), and a Duraloc titanium acetabular shell (DePuy) with an alumina ceramic liner. Given the shallow nature of the patient's native acetabulum, the decision was made to place the cup in the abduction angle (75°) that achieved the marriage between maximum degree of component coverage and joint stability. The patient made an uneventful postoperative recovery, and she was initially extremely pleased with the outcome of the surgery.

**Figure 1:**
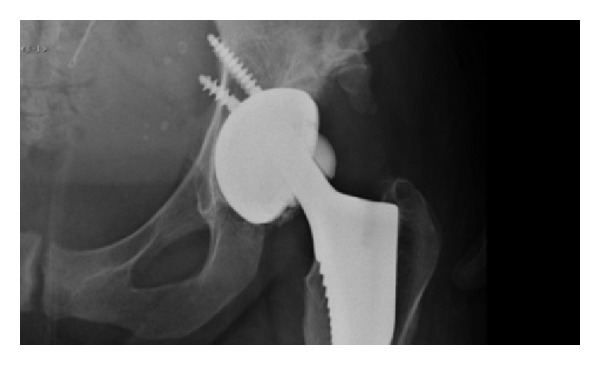
Radiograph demonstrating subtle fragmentation at the inferior head/neck junction.

**Figure 2:**
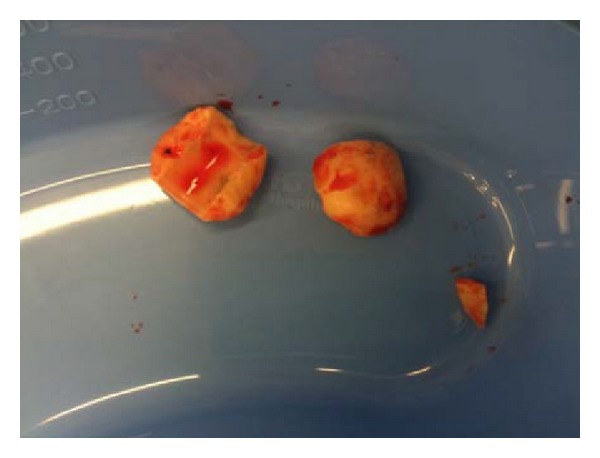
Ceramic head fragments removed at first revision.

Approximately twenty months after her primary hip replacement, the patient noticed that her hip began to squeak intermittently. A few months later, she was participating in moderate, low impact exercise when she experienced discomfort and a grinding sensation arising from her hip. Two days later, whilst turning over in bed, she felt significant pain and the sensation of the hip dislocating. The patient was admitted to hospital after X-rays demonstrated fragmentation of the ceramic femoral head (Figure 1). An urgent operative exploration of the hip was performed, revealing a fractured femoral head and a deeply scratched acetabular ceramic liner. The scratched liner was explanted, the fractured head fragments were removed (Figure 2), and a thorough lavage and debridement of the operative field was performed to oust the ceramic debris. The stem and cup were well fixed and hence left in situ. The liner was renewed with a 28 mm internal diameter “ceramic insert for metallic cup” (DePuy International Ltd., Leeds, England) and a Biolox Delta Articuleze ceramic 28 mm diameter, +1.5 neck length, 12/14 tapered cone head (DePuy Orthopaedics Inc., Warsaw, IN, USA) was applied to the trunnion of the stem as before. As with the primary procedure, the patient made an uneventful postoperative recovery. Whilst the patient's hip functioned well after the revision procedure, at subsequent outpatient based clinical reviews, the patient noted that her hip continued to squeak intermittently.

**Figure 3:**
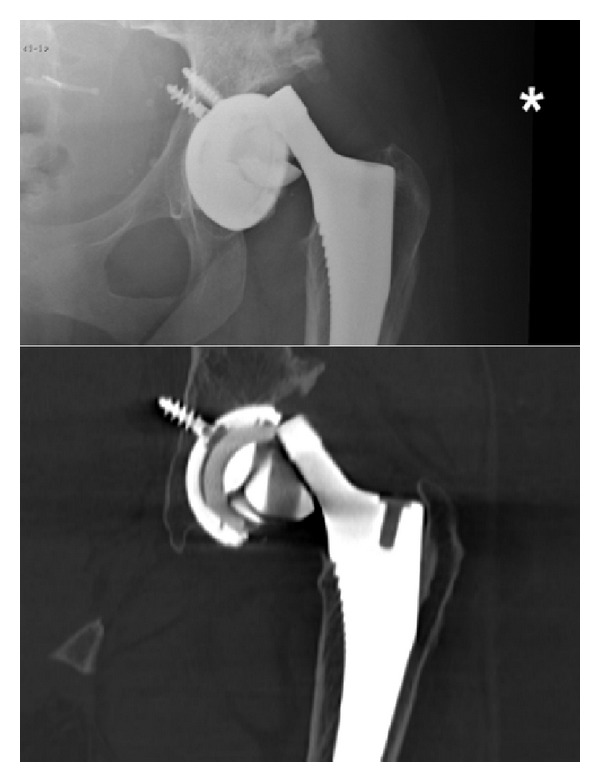
Plain radiograph and CT scan demonstrating fragmentation and dislocation of the ceramic head.

**Figure 4:**
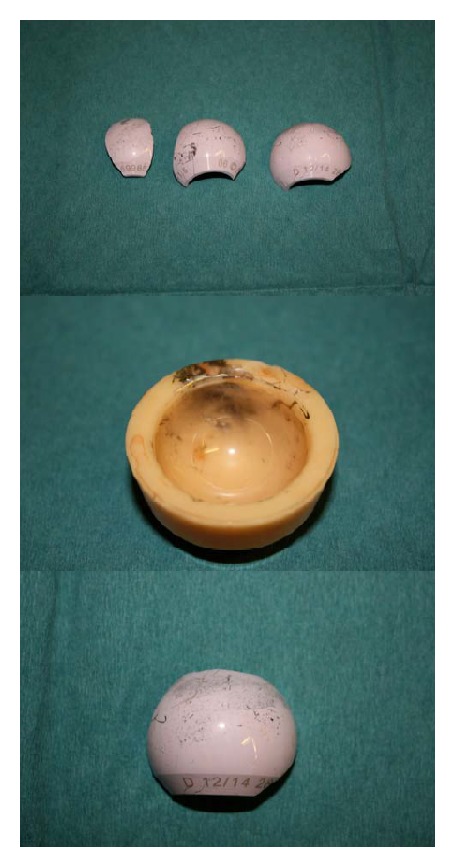
The ceramic components removed at the second revision procedure.

**Figure 5:**
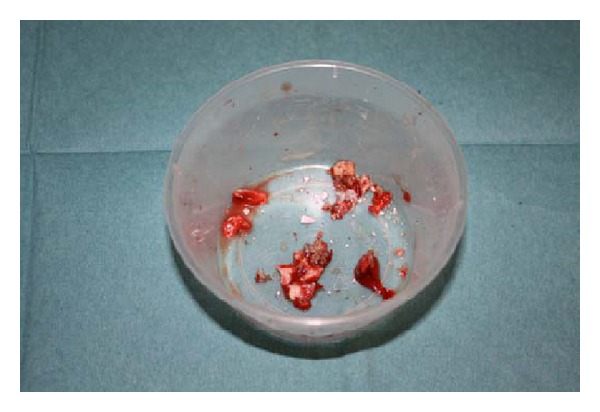
Ceramic debris excised from the soft tissues.

**Figure 6:**
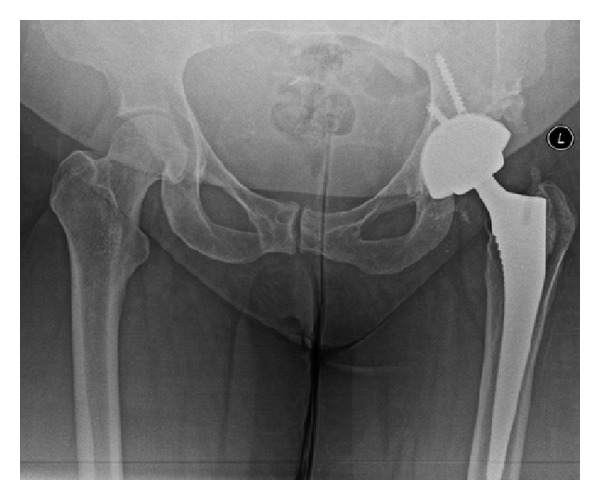
Acetabular component revised to a less abducted position. Ceramic bearings changed and trunnion sleeve utilized.

Six years following her revision procedure, the patient was again taking part in moderate, low impact exercise, when she felt a severe pain in her hip, with the sensation of the hip dislocating. Subsequent emergent radiographic investigations demonstrated a fractured ceramic head (Figure 3). After communication with the regional tertiary referral centre (where the index procedure had been performed), the patient was taken to theatre. Here, the ceramic liner was explanted together with the ceramic head fragments (Figure 4). The original metal acetabular shell was explanted and replaced with a 54 mm Delta TT cup (Lima Corporate Spa, Via Nationale, Udine, Italy). The cup fixation was supplemented with two 6.5 mm screws. A large (36 mm) Biolox delta ceramic liner was utilized (Lima Corporate Spa, Udine, Italy). The trunnion of the well-fixed femoral component was noted to be damaged. A metallic Bio-Ball trunnion sleeve (12/14, −3.0 mm) was positioned over the scratched trunnion, and a 36 mm Bio-Ball delta ceramic head was inserted into the trunnion sleeve (Bio-Ball; Merete, Berlin, Germany). A thorough lavage and debridement of the operative field was performed in an attempt to remove all of the ceramic debris (Figure 5). The immediate postoperative recovery was uneventful, and the patient was discharged home at day 7 postoperatively (Figure 6). At the time of writing, 5 months after the second revision procedure, the patient was walking independently, with good hip function and with no further squeaking emanating from the hip joint.

## 3. Discussion

Ceramic-on-ceramic articulations possess a number of advantageous characteristics, including superb wear and scratch resistance, hardness, and high wettability. These benefits may explain the increase in the use of ceramic bearings over the last ten years [5]. However, ceramics demonstrate virtually no ductility and hence their intrinsic brittle nature means that the potential for catastrophic failure due to fracture exists. Ceramic bearings have been used in total hip arthroplasty for over 30 years [2], and early designs were associated with component fracture [6]. However, with hot isocratic pressing, laser marking, and proof-testing, fracture of third generation ceramics is very rare [2], with reported rates between 0 and 0.004% [7]. Hence, the occurrence of a recurrent ceramic femoral head fracture in the same individual's total hip replacement is even rarer. Thus, we believe this case can be utilized to highlight potential risk factors for ceramic bearing fracture.

Ceramic bearings demonstrate very little ductility and hence may be prone to fracture when subjected to impact loading. Their high modulus of elasticity results in an intolerance to nonuniform loading. This may explain why acetabular components inserted in an excessively high degree of abduction (>55°) may demonstrate edge loading and have high wear rates when compared with components positioned optimally [8]. Therefore, it may be reasonable to propose that cup positioning can affect the likelihood of catastrophic ceramic failure. This theory is reenforced by our case, where the inclination angle of the primary acetabular component is measured at 75°. At the initial revision operation, the decision was made to leave the well-fixed acetabular component in situ but replace the scratched ceramic liner. At the second revision, however, despite the cup remaining well fixed, it was explanted, and a new cup was inserted in a more closed position compared with the primary component. Perhaps revising the cup after the first ceramic fracture would have prevented the second failure. In contrast to this theory, however, no link has been established between a high cup inclination angle and ceramic head fracture and, therefore, it is debatable as to whether adjusting the cup position at the first revision procedure would have prevented the second ceramic head fracture.

The head bore is a tapered hole created in a modular femoral head into which the Morse taper of the femoral component is inserted. The distance between the corner of the bore and the external surface of the ceramic head is less in short-neck tapers when compared with medium or long-neck tapers. In our case report, the ceramic head had a neck length of +1.5 mm in both the index and revision procedures. This observation may suggest that the risk of ceramic head fracture is increased when short-neck taper components are utilized. This theory is reenforced by a multicentre retrospective study of 312 patients who underwent ceramic-on-ceramic total hip arthroplasty [9]. In this cohort, 5 patients suffered ceramic head fractures, with all 5 individuals having a short-neck ceramic head in situ. Of interest, the pattern of head fracture demonstrated in our case (Figure 3) is identical to that in the study by Koo et al., involving the thinnest circumferential portion of the head, adjacent to the proximal edge of the head bore, with vertical cracks extending from the circular crack to the lower edge of the head component.

Stripe wear is a phenomenon that describes a localized crescent-shaped surface discoloration on the ceramic head. This was demonstrated on the head fragments excised at the second revision procedure and a reciprocal discolouration was apparent on the superior aspect of explanted ceramic liner (Figure 3). One theory concerning the aetiology of stripe wear is the development of high contact stresses at the articulating surfaces as a result of an abducted cup position. Another reported theory is that of microseparation of the bearing surfaces during the gait cycle. Large separations of the articulating surfaces may result in high-impact loads and the risk of considerable damage to the ceramic [8]. Perhaps this could be in keeping with our patient developing symptoms whilst performing moderate exercise.

Our patient noticed squeaking of both her primary and initial revision prostheses. Each of these went on to fail catastrophically. Squeaking in ceramic-on-ceramic hip bearings is likely to be multifactorial and related to patient factors (younger, heavier, and taller patients), surgical factors, and component factors (malpositioning of the acetabular shell) [10]. Currently, however, there is no evidence to suggest that the development of squeaking in a ceramic bearing hip joint indicates impending component fracture.

## 4. Strategies in Management of Ceramic Component Fracture

Revision surgery should not be delayed, as ceramic debris is likely to remain in the joint and could potentially affect either femoral or acetabular components, resulting in metallosis or bone destruction [11]. Once a radical synovectomy and capsular excision have been performed, options at the time of revision surgery for ceramic fracture include replacement of the bearings with a metallic head and polyethylene liner, revision of the stem and head, or insertion of a new ceramic head on the retained stem. Our clinical photographs demonstrate that ceramic fracture may result in the production of several large fragments, together with multiple smaller fragments and ceramic debris. Despite a radical synovectomy and thorough washout of the hip joint, it may be impossible to remove all of the minute ceramic fragments. This ceramic debris may subsequently become embedded in a polyethylene liner, cause rapid and substantial wear, and result in early failure [11]. Therefore, we believe one should avoid revising to metal-on-polyethylene articulations after ceramic component fracture.

Whilst placing a new ceramic head on an apparently undamaged trunnion has been shown to be effective, this may be against the advice of manufacturers and furthermore it is impossible to exclude microscopic trunnion damage at the revision operation. If the trunnion of the femoral component has been damaged by ceramic fragments or debris, insertion of a new ceramic head on the preexisting taper is not recommended [9, 12]. Areas of damage on a trunnion may result in a stress riser that could initiate and propagate a crack in the ceramic head, resulting in catastrophic failure [13]. As in our case, the use of a trunnion adaptor or sleeve may be considered and ensures a pristine interface between ceramic and metal. In a retrospective review of 126 revision total hip replacements, Jack et al. reported excellent rates of survival and function after utilizing a sleeve on a used/damaged trunnion together with a ceramic head [14]. Of note, in this study, no fractures occurred with the use of delta ceramics, whereas two alumina heads required revision for fracture.

The final option in the management of ceramic head fracture would be to revise the femoral stem in order to obtain a new, unused trunnion. However, surgeons may be reluctant to take this option, particularly with a well-fixed, cementless stem, due to the associated risks, which include increased operative blood loss and operating time, chronic pain, and damage to the proximal femur at time of explantation [13].

The strategies for approaching the revision of a ceramic-on-ceramic THA remain controversial. Based upon our experience from this case, coupled with a review of the relevant literature, we believe that a ceramic head fracture should be managed in the following way.

A radical synovectomy, capsular excision, and thorough irrigation should be performed in an attempt to remove as much ceramic debris as possible from the joint. Loose or malpositioned components, either femoral or acetabular, should be revised appropriately (cup inclination should be less than 55°). If a well-fixed stem is left in situ, regardless of the macroscopic appearance of the taper, a sleeve should be utilized together with new delta ceramic bearings. It is paramount to ensure that the femoral head and acetabular liner are both seated concentrically.
